# Attentional bias towards negative stimuli in healthy individuals and the effects of trait anxiety

**DOI:** 10.1038/s41598-020-68490-5

**Published:** 2020-07-16

**Authors:** Emilie Veerapa, Pierre Grandgenevre, Mohamed El Fayoumi, Benjamin Vinnac, Océanne Haelewyn, Sébastien Szaffarczyk, Guillaume Vaiva, Fabien D’Hondt

**Affiliations:** 1 Univ. Lille, Inserm, CHU Lille, U1172 - LilNCog - Lille Neuroscience and Cognition, 59000 Lille, France; 2 CHU Lille, Clinique de Psychiatrie, CURE, 59000 Lille, France; 3 Centre national de ressources et de résilience Lille-Paris (CN2R), 59000 Lille, France

**Keywords:** Emotion, Human behaviour

## Abstract

This study aimed to investigate the time course of attentional bias for negative information in healthy individuals and to assess the associated influence of trait anxiety. Thirty-eight healthy volunteers performed an emotional dot-probe task with pairs of negative and neutral scenes, presented for either 1 or 2 s and followed by a target placed at the previous location of either negative or neutral stimulus. Analyses included eye movements during the presentation of the scenes and response times associated with target localization. In a second step, analyses focused on the influence of trait anxiety. While there was no significant difference at the behavioral level, the eye-tracking data revealed that negative information held longer attention than neutral stimuli once fixated. This initial maintenance bias towards negative pictures then increased with increasing trait anxiety. However, at later processing stages, only individuals with the highest trait anxiety appeared to fixate longer on negative pictures than neutral pictures, individuals with low trait anxiety showing the opposite pattern. This study provides novel evidence that healthy individuals display an attentional maintenance bias towards negative stimuli, which is associated with trait anxiety.

## Introduction

Vision, like the other sensory systems, is constrained by its limited capacity of processing. Attention allows coping with this issue by selecting specific information within our rich and complex environment that will benefit from more elaborate processing and access to consciousness. The properties of the retina also constrain visual perception by limiting high-resolution processing to the fovea, which covers only the central 2° of visual angle. In naturalistic viewing, attentional selection relies on eye movements allowing to bring visual stimuli onto the fovea^1^. Overt attention is functionally coupled to, and share neural mechanisms with^2–4^, covert attention that improves perception at a specific visual location without gaze reorienting but also guides eye movements^5–10^. Attentional selection is also supposed to operate according to two complementary and interacting processes^11–14^. The reflexive or exogenous attention is a fast, bottom-up (stimulus-driven) process that selects stimuli according to their physical features or because they are novel or unexpected. The voluntary or endogenous attention is a slower, top-down (goal-driven) process selecting stimuli that are expected or relevant to current goals. It is also increasingly recognized that emotionally laden stimuli, given their strong adaptive significance, benefit from preferential processing over neutral stimuli^15,16^. The exact nature of the mechanisms responsible for this effect remains open to debate, in particular regarding the degree to which they differ from classical attention mechanisms^17,18^. However, evidence suggests that "emotional attention" also involves a balance between bottom-up and top-down processes^17,19^. The valence-related salience of a stimulus may thus depend on internal factors^20^, and there has been much interest regarding the link between anxiety and the special status of negative information^21,22^.

Cognitive models of anxiety posit that, even though effective detection of danger is crucial for survival, interindividual differences exist regarding the processing of threatening information^23–27^. Trait anxiety, which constitutes a vulnerability to affective disorders^28,29^, is supposed to be associated with a preferential selection of aversive stimuli over neutral cues so that individuals with high trait anxiety, unlike those with low trait anxiety, would present with attentional bias (AB) to threat^19,30, 31^. One of the most commonly used paradigms to assess AB for emotional information is the emotional dot-probe task^32^. In this task, two visual stimuli (one emotional stimulus and one neutral stimulus; either words, photographs of facial expressions, or pictures of natural scenes) are displayed simultaneously on the left and right side of a computer screen. AB towards or away from emotional stimuli is respectively inferred by faster or slower responses to detect a probe replacing an emotional stimulus than a probe replacing a neutral stimulus. The meta-analysis on threat-related AB performed by Bar-Haim et al.^30^, which included numerous dot-probe studies, found that individuals suffering from an anxiety disorder or with high trait anxiety, but not non-anxious persons, show an AB towards threat-related stimuli of comparable magnitude.

Two main hypotheses attempt to account for the influence of anxiety on visual attentional processing^33^. On the one hand, the "vigilance-avoidance" hypothesis^26^ suggests that anxiety is associated with facilitated orienting of attention towards threat-related stimuli. These vigilance responses, based on a reflexive selection of negative stimuli, are thought to be followed by a more strategic avoidance of this information. On the other hand, the "maintenance" hypothesis^34^ posits that threat-related stimuli, once they are detected, hold attention longer than neutral stimuli in anxious individuals. This attentional maintenance bias could be due to a general difficulty associated with trait anxiety in recruiting top-down control systems in situations involving competing stimuli and when task demands do not fully occupy attentional resources^19,24,35,36^. Several studies used emotional dot-probe task with different stimuli durations to investigate the time course of attentional allocation. The underlying rationale is that using different stimuli durations allows distinguishing between initial vigilance (short exposure duration) and later maintenance of attention (long exposure duration)^37–40^. The meta-analysis by Bar-Haim et al.^30^ found that anxious participants showed an AB towards negative information for all the tested exposure times (subliminal, 500 ms, or ≥ 1,000 ms), but differences with healthy controls were not significant for longer stimuli durations. Studies specifically designed to assess the time-course of AB for threatening stimuli with different exposure durations led to mixed results: while the study by Mogg et al.^38^ found no effect of exposure duration, others evidenced increased vigilance in individuals with high trait anxiety only for a short exposure duration (500 ms)^37,39,40^, sometimes followed by avoidance with increasing exposure time^40^. It appears, therefore, that AB for aversive stimuli operates at an early attentional stage, whereas evidence is less clear regarding the maintenance of attention.

Furthermore, there are many concerns expressed regarding the link between anxiety and disproportionate attention to aversive stimuli, and on the efficiency of AB assessment methods^41,42^. If some authors proposed that contradictory results obtained by dot-probe studies are due to methodological heterogeneity (e.g., the types of stimuli used or of anxiety investigated^43^), a recent meta-analysis did not found any evidence for AB towards threat in individuals with clinical anxiety (n = 1,005)^44^. This meta-analysis used another source of dot-probe data that emerged after the meta-analysis of Bar-Haim et al.^30^, namely baseline measures of AB obtained in randomized controlled trials evaluating the clinical efficacy of attentional bias modification procedures as a treatment for post-traumatic stress disorder (PTSD), social anxiety or panic disorder. It was thus not possible for the authors to test whether healthy individuals display an AB. Moreover, all the 13 studies included in this meta-analysis used a stimulus duration of 500 ms, leaving untested the possibility for AB to emerge after more prolonged exposure to stimuli. Finally, and importantly, one limitation of behavioral measures such as response time (RT) is that they only inform on the final processing output. In the context of dot-probe tasks, RT-based measures inform on attention at a single time point only, after stimuli replacement by the probe stimulus^45,46^ and, therefore, do not directly assess information processing during actual exposure to stimuli.

The eye-tracking technology automatically detects eye position and gaze direction with a high temporal resolution and is thus an interesting method to investigate the time course of attentional deployment. Eye movements are faster and considered as a more direct manifestation of attention than manual responses, so that they may be more adapted to the study of emotional attention^18,47^. Although eye-tracking studies on this topic are limited^18^, a series of studies using free viewing tasks found that healthy volunteers were more likely to fixate first on negative scenes presented concurrently with neutral scenes^46,48–51^. However, Quigley et al.^46^ observed this initial orienting bias towards negative stimuli regardless of trait anxiety, and Calvo and Avero^52^ only in individuals with high trait anxiety. Studies analyzing attentional maintenance also found mixed findings. Some authors reported that healthy volunteers showed a higher viewing time on negative pictures than on neutral pictures at the beginning of exposure^48^, regardless of trait anxiety^46^. Conversely, the study by Calvo and Avero^52^ revealed that only individuals with high trait anxiety showed higher gaze duration for pictures depicting actual harm than neutral pictures, followed by the opposite effect at later processing stages.

Given these controversial results, the present study used the eye-tracking technology to clarify (1) the time course of attentional capture by negative stimuli in healthy volunteers and (2) the associated influence of trait anxiety. To ensure comparability with previous behavioral studies investigating AB for negative stimuli, we used an emotional dot-probe task. Thirty-eight individuals took part in this study in which pairs of negative and neutral scenes selected from the International Affective Picture System^53^ (IAPS) were presented for either 1,000 or 2,000 ms and followed by a target placed at the previous location of either negative (i.e., congruent condition) or neutral stimulus (i.e., incongruent condition). The two exposure durations allowed to assess the dynamics of information processing with both analyses of eye movements during actual exposure to stimuli and standard AB measures calculated from RTs associated with correct probe detection. Regarding eye movements, analyses of first fixation direction and latency allowed to investigate the initial orientation of attention, whereas analyses of dwell time and the total count of fixations served as indexes of the overall maintenance of attention. We also examined the first fixation duration as an index of initial maintenance of attention, and the average duration of fixations as an index of re-engagement of attention. In a second step of the analyses, we investigated the links between trait anxiety, assessed with the trait part of the State-Trait Anxiety Inventory (STAI, Form Y)^54^, and eye-tracking measures, as well as RT-based measures. The influence of cognitive control is likely to increase as the visual information is processed, so trait anxiety effects would concern mostly maintenance of attention, and could be stronger with increasing exposure time^19,24,35,36^.

## Results

### Characteristics of the stimuli

The stimuli were pictures selected from the IAPS according to gender. Each gender-based set included 24 negative and 24 neutral pictures (see Table S1 in Supplemental Information). Regarding emotional characteristics, valence was significantly lower for negative pictures than for neutral pictures, and arousal was significantly higher for negative pictures than for neutral pictures (all *p* values < 0.05). Regarding visual characteristics, negative and neutral pictures did not differ in terms of luminance, color saturation (RGB), and energy across spatial frequencies (all *p* values > 0.05). Finally, regarding cognitive characteristics, negative and neutral pictures did not differ in terms of complexity (JPEG file size), and face area (the percentage of the area occupied by faces in each picture; all *p* values > 0.05).

### Trait anxiety

Among the 38 participants, the mean STAI score for trait anxiety was 41 ± 9 (range = 26–58). According to the French norms^54^, twelve participants had very low trait anxiety (T-note ≤ 35), 13 had low trait anxiety (T-note between 36 and 45), 12 had medium trait anxiety (T-note between 46 and 55), 1 had high trait anxiety (T-note between 56 and 65), and none had very high trait anxiety (T-note > 65).

### Eye-tracking data

The threshold of significance was set at *p* < 0.008 (see “Eye gaze data” section).

#### First fixation direction

On average, the first fixation occurred on one of the two pictures in 80 ± 16% of the trials. The probability of first fixating the negative pictures (M = 40.68%, SE = 1.47) did not significantly differ from 50%, t(37) = 0.83, *p* = 0.410, d = 0.14, suggesting that there was no significant orienting bias towards or away from negative stimuli. The probability of first fixating the negative pictures was not significantly correlated with trait anxiety, r = −0.19, *p* = 0.255.

#### First fixation latency

The first fixation latency did not significantly differ between the neutral pictures (Mdn = 238 ms) and the negative pictures (Mdn = 261 ms), V = 502, *p* = 0.057, r = −0.31. The difference between first fixation latencies for negative and neutral pictures was not significantly correlated with trait anxiety, r = 0.11, *p* = 0.525.

#### First fixation duration

The first fixations were significantly longer for the negative images (Mdn = 233 ms) than for the neutral images (Mdn = 206 ms), V = 589, *p* = 0.001, r = −0.53. The difference between first fixation durations for negative and neutral pictures was not significantly correlated with trait anxiety, r_s_ = 0.17, *p* = 0.301.

#### Dwell time

The means and standard errors of dwell time are shown in Table 1. There was a significant main effect of exposure duration, F(1,37) = 363.32, *p* < 0.001, η^2^_p_ = 0.91, showing longer dwell time in the 2,000 ms condition (M = 533 ms, SE = 24) than in the 1,000 ms condition (M = 240 ms, SE = 10). The main effect of valence was also significant, F(1,37) = 34.36, *p* < 0.001, η^2^_p_ = 0.48, indicating longer dwell time on negative pictures (M = 417 ms, SE = 19) than on neutral pictures (M = 357, SE = 17). The interaction between exposure duration and valence was not significant, F(1,37) = 0.04, *p* = 0.852, η^2^_p_ < 0.01.

**Table 1:** Mean (standard error) dwell time (in ms) as a function of valence and exposure duration.

	Exposure duration	
	1,000 ms	2,000 ms
Valence		
Negative	271 (12)	562 (26)
Neutral	210 (9)	504 (25)

**Figure 1:**
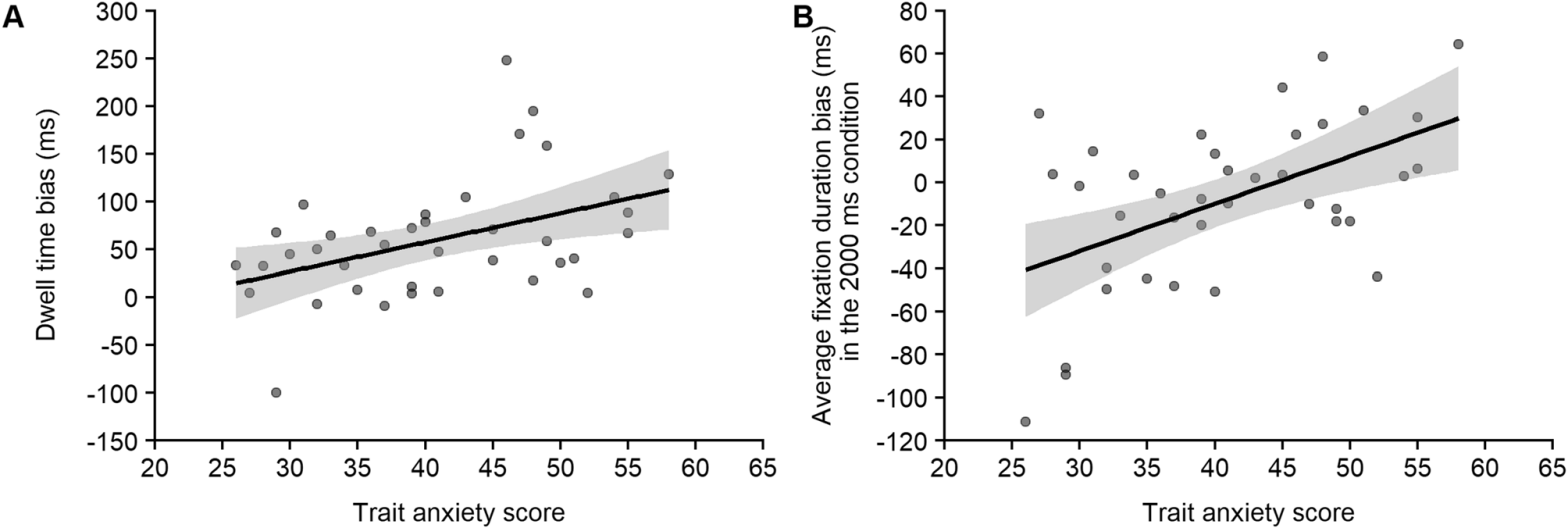
Scatterplots of the relationship between trait anxiety and (**A**) the dwell time bias (R^2^ = .19) and (**B**) the average fixation duration bias in the 2,000 ms condition (R^2^ = .26).

The addition of trait anxiety as a continuous predictor in the analysis of variance revealed that the main effect of trait anxiety, F(1,36) = 4.097, *p* = 0.051, η^2^_p_ = 0.10, the interaction between trait anxiety and exposure duration, F(1,36) = 4.92, *p* = 0.033, η^2^_p_ = 0.12, and the interaction between trait anxiety, exposure duration and valence, F(1,36) = 0.93, *p* = 0.342, η^2^_p_ = 0.03, were not significant. However, there was a significant interaction between trait anxiety and valence, F(1,36) = 8.31, *p* = 0.007, η^2^_p_ = 0.19 (Fig. 1A). Further analysis of the association between trait anxiety and valence revealed that the bias in dwell time towards negative pictures increased with increasing trait anxiety (see Supplementary Information, 1.1, for details).

#### Total fixation count

The means and standard errors of the total fixation counts are shown in Table 2. There was a significant main effect of exposure duration, F(1,37) = 264.24, *p* < 0.001, η^2^_p_ = 0.88, showing more fixations in the 2,000 ms condition (M = 2.347, SE = 0.102) than in the 1,000 ms condition (M = 1.283, SE = 0.048). The main effect of valence was also significant, F(1,37) = 41.27, *p* < 0.001, η^2^_p_ = 0.53, indicating more fixations on negative pictures (M = 1.971, SE = 0.084) than on neutral pictures (M = 1.660, SE = 0.068). The interaction between exposure duration and valence was not significant, F(1,37) = 3.49, *p* = 0.070, η^2^_p_ = 0.09.

**Table 2:** Mean (standard error) total fixation count as a function of valence and exposure duration.

	Exposure duration	
	1,000 ms	2,000 ms
Valence		
Negative	1.410 (0.056)	2.532 (0.120)
Neutral	1.157 (0.047)	2.162 (0.095)

The addition of trait anxiety as a continuous predictor in the analysis of variance revealed that the main effect of trait anxiety, F(1,36) = 4.17, *p* = 0.048, η^2^_p_ = 0.10, the interaction between trait anxiety and exposure duration, F(1,36) = 7.60, *p* = 0.009, η^2^_p_ = 0.17, the interaction between trait anxiety and valence, F(1,36) = 4.29, *p* = 0.046, η^2^_p_ = 0.11, and the interaction between trait anxiety, exposure duration and valence, F(1,36) = 0.00, *p* = 0.998, η^2^_p_ < 0.01, were not significant.

#### Average fixation duration

The means and standard errors of the average fixation duration are shown in Table 3. The main effect of valence was not significant, F(1,37) = 0.14, *p* = 0.714, η^2^_p_ < 0.01. The main effect of exposure duration was significant, F(1,37) = 66.65, *p* < 0.001, η^2^_p_ = 0.64, showing that average fixation duration was longer in the 2,000 ms condition (M = 249 ms, SE = 9) than in the 1,000 ms condition (M = 194 ms, SE = 5). The interaction between exposure duration and valence was not significant, F(1,37) = 5.66, *p* = 0.023, η^2^_p_ = 0.13.

**Table 3:** Mean (standard error) average fixation duration (in ms) as a function of valence and exposure duration.

	Exposure duration	
	1,000 ms	2,000 ms
Valence		
Negative	199 (5)	245 (10)
Neutral	188 (6)	253 (10)

The addition of trait anxiety as a continuous predictor in the analysis of variance did not reveal any significant main effect of trait anxiety, F(1,36) = 0.42, *p* = 0.522, η^2^_p_ = 0.01. The interaction between trait anxiety and exposure duration, F(1,36) = 0.29, *p* = 0.591, η^2^_p_ < 0.01, and the interaction between trait anxiety and valence, F(1,36) = 4.67, *p* = 0.037, η^2^_p_ = 0.11, were not significant. There was a significant interaction between trait anxiety, exposure duration and valence, F(1,36) = 10.61, *p* = 0.002, η^2^_p_ = 0.23. The difference in average fixation duration between negative pictures and neutral pictures was significantly correlated with trait anxiety in the 2,000 ms condition (r = 0.51, *p* = 0.001; Fig. 1 B) but not in the 1,000 ms condition (r = −0.13, *p* = 0.422). Further analysis of this significant association in the 2,000 ms condition revealed that, while average fixation duration was shorter for negative pictures than for neutral pictures among individuals showing the lowest trait anxiety scores, this bias was reversed and not significant among individuals showing the highest trait anxiety scores (see Supplementary Information, 1.1, for details).

### Response times

The threshold of significance was set at *p* < 0.05. AB scores (i.e. the difference of mean RT between congruent and incongruent trials) were not significantly different from 0 in the 1,000 ms condition (M = −2 ms, SE = 4), t(37) = −0.38, *p* = 0.703, d = −0.06, and in the 2,000 ms condition (M = −1 ms, SE = 4), t(37) = −0.33, *p* = 0.745, d = −0.05. There was no significant main effect of exposure duration, F(1,37) = 0.00, *p* = 0.974, η^2^_p_ < 0.01. The addition of trait anxiety as a continuous predictor in the analysis of variance did not reveal any significant effect of trait anxiety, F(1,36) = 1.50, *p* = 0.228, η^2^_p_ = 0.04, or significant interaction between trait anxiety and exposure duration, F(1,36) = 0.64, *p* = 0.430, η^2^_p_ = 0.02.

## Discussion

The objective of this study was twofold, as it aimed to (1) investigate the time course of attentional capture by negative stimuli in healthy volunteers and (2) assess the associated influence of trait anxiety. To this end, we recorded eye movements and RTs of 38 healthy participants performing an emotional dot-probe task in which pairs of negative and neutral scenes were presented simultaneously for either 1,000 or 2,000 ms.

Previous results from eye-tracking studies in which participants viewed pairs of emotional and neutral scenes were controversial. Regarding initial orienting of attention, some studies showed more first fixations on negative pictures than neutral pictures^46,48–51^, regardless of trait anxiety^46^, or only in high trait anxious individuals^52^. Regarding attentional maintenance, some studies reported a higher viewing time on negative pictures than on neutral pictures at early processing stages in healthy volunteers^48^, regardless of trait anxiety^46^. Others found this effect only in individuals with high trait anxiety, which then avoided aversive stimuli^52^.

In our study, analyses of eye-tracking data did not reveal any significant difference between negative and neutral stimuli regarding first fixation direction and first fixation latency, even when considering trait anxiety. Nonetheless, we found both higher dwell time and more frequent fixations on negative pictures than on neutral pictures, regardless of stimuli duration. From these results, it appears that healthy volunteers presented, therefore, with an attentional maintenance bias towards aversive stimuli in the first 1,000 ms of exposure, and not in the last 1,000 ms of exposure. Moreover, the analysis of dwell time also revealed that the AB increased with increasing anxiety. Altogether, results are thus in favor of the attentional maintenance hypothesis^34^, which posits, unlike the vigilance-avoidance hypothesis^26^, that there is no facilitation of attentional orienting towards threat-related stimuli, which hold attention once detected^33^.

However, the results obtained for the first fixation duration and the average fixation duration refined the analysis of the dynamics of AB to negative pictures. The first fixation duration was longer on negative stimuli than on neutral stimuli, regardless of trait anxiety. Therefore, higher trait anxiety increased the dwell time bias towards negative pictures in the first 1,000 ms of exposure, but probably only after the first fixation on one of the two pictures. Moreover, regarding the average fixation duration, the difference between negative and neutral pictures correlated with trait anxiety only in the 2,000 ms condition. This significant association showed that, at later processing stages (i.e., during fixations in the last 1,000 ms of the 2,000 ms condition), neutral stimuli held attention longer in individuals with low trait anxiety. In contrast, negative pictures still tended to hold longer attention in more anxious individuals.

Interestingly, the model of Williams et al.^27^ posits that trait anxiety influences the allocation of attention after the assessment of the threat value of a stimulus. According to this model, individuals with high trait anxiety would be more likely to orient towards aversive stimuli, while individuals with low trait anxiety may present a tendency to shift their attention away from these stimuli. From our results, we can suggest that predictions of this model and that of the maintenance hypothesis are not incompatible and may reflect different attentional stages. Indeed, it appears that negative information holds longer attention than neutral stimuli once fixated. In line with the maintenance hypothesis, this initial maintenance bias increases with increasing trait anxiety, but only transiently. Then, in line with the model of Williams et al.^27^, only individuals with the highest trait anxiety longer fixate negative pictures than neutral pictures, whereas individuals with low trait anxiety present a maintenance bias away from aversive information.

Importantly, the interpretation in terms of attentional maintenance implies that higher dwell time and longer fixation duration reflect sustained attention, as classically hypothesized by eye-tracking studies on attention^18,47^. First, several bottom-up and top-down factors are likely to influence gaze fixation and duration^55^. For instance, some categories of visual stimuli can attract more fixations than others because of their visual saliency. In line with previous works^48–50^, we carefully controlled the physical parameters of images to minimize the possibility that these stimulus-driven factors influenced our results. Besides, longer dwell time or fixation duration can also reflect processing difficulty^56^. For this reason, we also considered pictures' complexity, in line with previous studies using pictures of natural scenes^48,49,51^, so that the only significant difference between the negative pictures and the neutral pictures concerned their affective dimensions.

Then, in eye-tracking research, the choice of the task is critical to draw valid conclusions as it determines inferences regarding the cognitive processes responsible for the eye movements^57^. On the one hand, in line with previous eye-tracking studies investigating attention to pairs of emotional and neutral images, we used a free viewing task with fixed stimuli duration^46,48–52^. Fixed exposure duration induces time pressure but can also create experiences of idleness that could significantly influence the interpretation of data^55,58^, in particular when participants have no instruction at all. One can assume that it is most likely more evident to link eye movements to the attentional allocation when free viewing tasks require a response as they encourage participants to be attentive^59^. The difference of paradigm may thus explain the discrepancies between the eye-tracking results obtained when participants had no specific instructions^46^ and results of studies requiring, like ours, a manual response by the participants^48–52^.

On the other hand, however, the relevance of the stimuli for the task is another critical factor to consider as it influences the interpretation of data^55,60^. Regarding eye-tracking research on attentional capture by emotional stimuli, tasks with instructions emphasizing the affective content of pictures imply looking at both pictures. In the previous studies mentioned earlier, participants had to discriminate valence of pictures^48,51,52^, or determine whether a subsequently presented picture had the same content as one of these two scenes^49,50^. Accordingly, the emotional content of the stimuli was relevant to the task and probably influenced the time course of information processing. In this context, it is thus challenging to determine whether valence-related differences in eye-tracking measures in either early or late processing stages reflected effects in initial orienting or maintenance of attention. For instance, the study by Calvo and Lang^48^ found that the emotional advantage was limited to early attentional maintenance. In their study, however, the lack of significant difference between emotional and neutral pictures at later processing stages was possibly due to the task as participants were required to determine whether the valence of the two concomitantly presented pictures differed or not. Thus, participants, after having oriented first their attention towards the emotional stimulus, probably looked at the neutral picture to perform the task accurately. In the study by Calvo and Avero^52^, the same task was employed and may have limited the possibility to investigate some anxiety effects at later time intervals^36^.

Contrary to those studies, we employed the dot-probe paradigm, which is classically used to assess selective attention^32^. Participants had to detect targets appearing (1) just after the natural scenes that were presented during an unpredictable duration of either 1,000 ms or 2,000 ms and (2) at an unpredictable location, either the one previously occupied by the negative stimulus or the one previously occupied by the neutral stimulus. Using this paradigm, we probably assessed more directly the influence of emotion on the spatio-temporal deployment of visual attention than studies with free viewing tasks without specific instruction or requiring valence discrimination, or a subsequent recognition test^46,48–52^. Furthermore, these different tasks probably involve different attentional mechanisms^17,46^, which could explain while we failed to find any evidence for a preferential initial orienting towards negative pictures, unlike those studies. Indeed, even though orienting towards emotional stimuli may be exogenously controlled, there is evidence than top-down factors also influence first fixations on emotional pictures. For example, participants can saccade to neutral pictures rather than emotional pictures through explicit instructions^51^. Exogenous saccades are also supposed to be faster than the ones observed in those studies (< 200 ms)^61,62^. One possibility is, therefore, that preferential initial orienting towards negative pictures imply that these stimuli are relevant to current goals. Another possible influencing factor is the first fixation direction bias for the left hemifield, described in the literature in association with reading and writing habits^63,64^. In agreement with this idea, further analysis of the first fixation direction in the present study (see Supplementary Information, 1.2, for details) revealed that the probability of first fixating on the picture in the left hemifield was higher than the probability of first fixating on the picture in the right hemifield. This left hemifield bias may have contributed to lowering the validity of this index to measure initial orientation as a function of picture content.

Even though the present eye-tracking study is, to the best of our knowledge, the first to use a dot-probe task with natural scenes, a few others eye-tracking studies have used a dot-probe task with facial stimuli, in the context of social anxiety^59,65^, social phobia^66^, generalized anxiety disorder^67^, or PTSD^68^. One study focusing on initial orienting of attention found that high trait anxious individuals were more likely to fixate, first, negative faces than neutral faces^69^. One possible explanation is that, because facial stimuli have greater intrinsic biological relevance than natural scenes, they are more prone to trigger initial vigilance responses^18^. Conversely, pictures of natural scenes would be more likely to warrant maintained attentional processing due to their greater complexity^39^. This interpretation remains hypothetical, and further studies should investigate this issue.

The analysis of RT data in the emotional dot-probe task did not reveal any significant AB for negative information, which was also not significantly correlated with trait anxiety. These results support the idea that RT-based measures do not index AB for aversive stimuli in healthy volunteers. One could assume that these results are in line with the previous literature^30^ as our sample was mainly composed of individuals presenting with low to moderate trait anxiety, and we used longer stimuli durations. However, because eye-tracking results provided a different picture, they can also suggest that classic RT-based measures in dot-probe tasks do not constitute reliable indexes of AB^70,71^. We cannot exclude either that both RT-based and eye-tracking results regarding the link between trait anxiety and attentional bias would differ for individuals with higher trait anxiety. Future studies should investigate this issue.

It has been argued that it is difficult to differentiate between covert and overt attentional shifts from the RT-based results of emotional dot-probe studies, as most of these studies did not use specific gaze behavior instructions^72^. The present study measured eye movements, i.e. overt attention, but we cannot exclude that AB to negative stimuli also implies covert attentional shifts. For instance, a maintenance bias towards negative stimuli might occur before the initial fixation^47,59^. Considering that eye movements follow covert attentional shifts^6^, greater engagement in, or difficulty in disengaging from, negative stimuli may lead to longer first fixation latency for unpleasant pictures than neutral pictures. However, the difference of first fixation latency between negative and neutral pictures failed to reach significance in the present study (*p* = 0.057) and was not significantly correlated to trait anxiety. Besides, eyes are in constant motion even when people fixate one specific point in visual space. Interestingly, and despite controversial data^73^, it is supposed that the direction of microsaccades, which can occur up to three times per second during fixation^74^, can index covert attentional shifts^75,76^. Future works measuring eye movements to assess AB to emotional stimuli should consider investigating the direction of microsaccades during fixations to infer covert attentional shifts using the adequate eye-tracking methodology^77^.

Finally, further research should also consider other relevant variables, notably the age of participants. The present study recruited young adults, and AB towards negative stimuli is likely to be different or even nonsignificant with older adults^78^. Future studies should try to determine whether anxiety disorders and PTSD are associated with specific patterns of eye movements using the procedure employed in this study. This research would pave the way for the development of new treatments improving the most common procedures based on a modification of the visual dot-probe task^79^, the efficacy of which is currently debated^80,81^.

In conclusion, the present study provides new evidence that the emotional content of stimuli influences the time-course of visual attention, in part as a function of trait anxiety. The attentional bias for negative stimuli occurs after the initial orienting of attention, then transiently increases with increasing trait anxiety, and appears to persist longer only in individuals with high trait anxiety, individuals with low trait anxiety instead showing an attentional maintenance bias away from negative pictures at later processing stages.

## Methods

### Participants

Thirty-eight participants (mean age = 23 ± 3 years; 26 women) provided informed consent before participating in the present study, which was approved by the local ethics committee (Comité de protection des personnes Nord Ouest IV, France) and was conducted in accordance with the Declaration of Helsinki. All patients had normal or corrected-to-normal vision and lacked any neurological or psychiatric disorders or drug consumption (as assessed by a trained psychiatrist during an exhaustive interview). All had a score on the Montreal Cognitive Assessment (MoCA) test^82^ of 26 or above. Thirty-six participants were right-handed and 2 were left-handed^83^.

### Stimuli

Given the differences usually observed between men and women in the processing of emotional stimuli^84,85^, negative and neutral pictures from the IAPS^53^ were selected according to gender. Each gender-based set included 24 negative stimuli, including pictures associated with death and depicting attacks or injured, afraid or suffering persons, and 24 neutral stimuli, including pictures of inanimate objects or depicting people with neutral expressions or in neutral situations (such as daily activities for instance). Emotional characteristics of the stimuli were assessed from mean values of standardized IAPS ratings for valence (scale of 0–9, in which 0 indicates a very unpleasant picture and 9 indicates a very pleasant picture) and arousal (scale of 0–9, in which 0 indicates very calm and 9 indicates very arousing). Regarding visual characteristics, we computed for each picture average (and standard deviation values, as an index of contrast) luminance and RGB saturation values. Moreover, energy across spatial frequencies was assessed by using the MATLAB script provided by Delplanque et al.^86^, which separates the different layers (RGB) and performs a grayscale transformation of the pictures. Then, discrete wavelet analysis is performed with Haar discrete bidimensional orthogonal wavelets (eight levels). This transformation provides wavelet coefficient matrices for each level. The energy measure is obtained by averaging the sum of the squared values for these coefficients. Finally, regarding cognitive characteristics, we assessed the complexity of the pictures by considering the JPEG file size (number of kilobytes) and face area by computing the percentage of the area occupied by faces in each picture^48^. Negative and neutral scenes were tested for differences regarding these emotional, visual, and cognitive factors using Welch’s t-tests for independent samples or Wilcoxon rank-sum tests (when normality assumption was not met). The angular size of the images was 8° (horizontal) × 6° (vertical) at a fixed viewing distance of 1 m, and the distance between the fixation cross and the center of each image was 8°. The pictures were displayed on a gray background.

### Apparatus

The stimuli were displayed using a 22″ monitor (AOC, resolution: 1,680 × 1,050; refresh rate: 60 Hz) connected to a PC with an Intel Core i3-2,120 3.30 GHz processor and 3 GB RAM as well as an AMD Radeon HD5450 card. The presentation of stimuli and the recording of responses were performed using the Psychophysics Toolbox Version 3^87^ for MATLAB (R2015a, The MathWorks, Inc., Natick, Massachusetts, United States). Eye movements were recorded using the iViewX Hi-Speed eye tracker from Senso-Motoric Instruments (Teltow, Germany; connected to a PC with an Intel Pentium 4 3.00 GHz processor and 1 GB RAM) at a sampling rate of 350 Hz. The manufacturers report a gaze position accuracy of 0.25°–0.5°.

### Procedure

Upon arrival at the study, participants provided informed consent and completed the trait part of the STAI^54^. The participant was then presented with a central white square (40° × 40°) containing five calibration points. The participant was asked to fixate on the black dots (center, top right, top left, bottom right, and bottom left) while his/her eye positions were recorded by the system. Once the calibration was completed, the participant started the dot-probe task. A trial was triggered when fixation had been stable for 500 ms. Each trial began with a black fixation cross presented for a randomly selected duration between 500 and 1,500 ms on a gray background, followed by two stimuli, one neutral scene and one negative scene, presented simultaneously for a duration of either 1,000 ms or 2,000 ms (with an equal number of presentations for each presentation duration). A small black dot was randomly presented at the location previously occupied by the negative image (congruent trials) or by the neutral image (incongruent trials). Participants were instructed to focus their gaze on the central cross, then to freely explore the images and finally to indicate as quickly as possible the spatial location (left or right) of the dot by pressing one of two keys. A new trial started immediately after they gave their response. The experiment consisted of one practice block of 10 trials followed by one experimental block of 96 trials (24 pairs × 2 exposure durations × 2 trial types). Trials were presented in a randomized order.

### Data preparation

#### Eye gaze data

Eye-tracking data were analyzed using BeGaze software from SensoMotoric Instruments (Teltow, Germany). Areas of interest corresponding to the location of the negative image and the neutral image were identified and used to determine fixation locations and fixation durations. Only fixations longer than 80 ms were considered in the analyses. The initial orientation of attention was assessed using two indices for each picture type: (1) first fixation direction*,* which corresponded to the total number of trials in which the first fixation was made on the negative picture type divided by the total number of trials in which the first fixation was made on either the negative picture or the neutral picture, and (2) first fixation latencies. Initial maintenance of attention was assessed by analyzing first fixation durations for each picture type. The overall maintenance of attention was assessed using three indices computed for each exposure duration and picture type: (1) dwell time (i.e., overall gaze duration) served as a global index, (2) total fixation count was considered a reorientation index and (3) average fixation duration was considered a re-engagement index.

#### Response times

RTs corresponded to the time between the presentation of the dot and the button press. Trials with response errors were excluded (0.47% of data). No trial was excluded because of a very fast response time (all RTs ≥ 260 ms). However, some trials with long RTs (more than 2,000 ms) were removed (0.25% of data). Then, trials with RTs more than 2.5 SDs above or less than − 2.5 SDs below the participant's mean were discarded to reduce the influence of outliers (1.89% of data). Individual mean RT was computed for each experimental condition on the remaining trials (representing, on average, 97 ± 2% of the total number of trials). AB was computed for each individual and each exposure duration by subtracting the RT in incongruent conditions from the RT in congruent conditions.

### Data analysis

The statistical analyses were performed using the software R studio version 1.2.1335 (RStudio, Inc.).

#### Eye gaze data

Regarding our first objective, six statistical tests were performed, and statistical significance was accepted at a Bonferroni-adjusted alpha level of 0.008. Paired t-tests (or Wilcoxon signed-rank tests when normality assumption was not met) were performed to determine whether first fixation latencies and first fixation durations differed between negative and neutral pictures. A one-sample t-test was performed to determine whether the probability of first fixation on the negative picture significantly differed from 50 (a probability higher or lower than 50 indicates a bias towards or away from negative stimuli, respectively). Repeated measures analyses of variance (ANOVA) were performed on dwell time, total fixation count, and average fixation duration with valence (negative, neutral) and exposure duration (1,000, 2,000) as within-subject factors. Significant interactions were followed by pairwise comparisons. For these comparisons, statistical significance was accepted at a Bonferroni-adjusted alpha level of 0.004.

Regarding our second objective, six statistical tests were also performed, and like for the first objective, statistical significance was accepted at a Bonferroni-adjusted alpha level of 0.008. Links with trait anxiety were assessed by: (1) performing Pearson (or Spearman when normality assumption was not met) correlation analyses between trait anxiety and the probability of first fixation on the negative pictures, as well as the difference scores between negative and neutral conditions for first fixation latencies and first fixation durations; (2) by adding trait anxiety as a continuous predictor into a second step of the analysis of variance for other variables. In the case of a significant interaction between trait anxiety, exposure duration, and valence, Pearson correlation analyses were conducted between trait anxiety and difference scores according to valence at each exposure duration. For these analyses, statistical significance was accepted at a Bonferroni-adjusted alpha level of 0.004.

#### Response times

For all analyses, the threshold of significance was set at *p* < 0.05. One-sample t-tests were performed to determine whether AB measured from RTs in the 1,000 ms and 2,000 ms conditions significantly differed from 0. Then, a repeated-measures analysis of variance (ANOVA) was performed on AB measured from RTs with exposure duration as a within-subject factor. Link with trait anxiety was assessed by adding trait anxiety as a continuous predictor into a second step of the analysis of variance.

## Supplementary information

Supplementary information.

## Data Availability

The datasets generated during the current study are available from the corresponding author on a reasonable request.
