# Supplementary material for: Attentional bias towards negative stimuli in healthy individuals and the effects of trait anxiety
Source: Sci Rep. 2020 Jul 16;10:11826. doi: 10.1038/s41598-020-68490-5 (PMC7367300; doi:10.1038/s41598-020-68490-5)
Supplement: Supplementary file 1 — Supplementary information. [file 41598_2020_68490_MOESM1_ESM.doc]

# Attentional bias towards negative stimuli in healthy individuals and the effects of trait anxiety

Emilie **Veerapa**^a,b^, Pierre **Grandgenevre**^a,b^, Mohamed **El Fayoumi**^a^, Benjamin **Vinnac**^a^, Océanne **Haelewyn**^a^, Sébastien **Szaffarczyk**^a,b^, Guillaume **Vaiva**^a,b,c^, Fabien **D'Hondt**^a,b,c^,*

^a^ Univ. Lille, Inserm, CHU Lille, U1172 - LilNCog - Lille Neuroscience & Cognition, F-59000 Lille, France

^b^ CHU Lille, Clinique de Psychiatrie, CURE, F-59000 Lille, France

^c^ Centre national de ressources et de résilience Lille-Paris (CN2R), F-59000 Lille, France

* Correspondence should be sent to Fabien D'Hondt:

E-mail address: fabien.d-hondt@univ-lille.fr

Postal address: CURE, Service de psychiatrie de l'enfant et de l'adolescent, Hôpital Fontan 1, CHU de Lille, CS 70001, 59037 Lille cedex France

# Supplementary information

# 1. Results

## 1.1 Links between trait anxiety and attentional maintenance

To further explore the link between trait anxiety and the valence effect on dwell time and on average fixation duration in the 2000 ms condition, a median split was conducted on trait anxiety. The median for trait anxiety was 40. Two participants with a trait anxiety score of 40 were removed from the analysis. Those participants with a score lower than 40 were included in the "low trait anxiety" (LTA) group (n=18; trait anxiety: M = 33, SD = 4), and those with a score higher than 40 were included in the "high trait anxiety" (HTA) group (n=18; trait anxiety: M = 49, SD =5). Groups did not differ regarding age (LTA: M = 23, SD = 3; HTA: M = 23, SD = 3), W = 164.5, p = .950, or sex ratio (LTA: 12 women; HTA: 13 women), χ²(1) = 0.13, p = .717.

A Welsh's t-test for independent samples revealed that the dwell time bias (i.e., the difference in dwell time between negative pictures and neutral pictures) was significantly greater in the HTA group (M = 87.81, SE = 16.23) than in the LTA group (M = 29.22, SE = 10.47), t(29.06) = 3.03, p = .005, r = .49. One-sample t-tests showed that the dwell time bias was significantly greater than 0 in the LTA group, t(17) = 2.79, p = .006 (one-sided), d = 0.66, and in the HTA group, t(17) = 5.41, p < .001 (one-sided), d = 1.28.

A Welsh's t-test for independent samples revealed that the average fixation duration bias (i.e., the difference in average fixation duration between negative pictures and neutral pictures) in the 2000 ms condition significantly differed between the LTA group (M = -26, SE = 9) and the HTA group (M = 10, SE = 7), t(30.76) = 3.11, p = .004, r = .49. One-sample t-tests showed that the average fixation duration bias was significantly lower than 0 in the low trait anxiety group, t(17) = -2.71, p = .007 (one-sided), d = -0.64, and was not significantly greater than 0 in the high trait anxiety group, t(17) = 1.55, p = .070 (one-sided), d = 0.37.

## 1.2 First fixation laterality

Because of the literature suggesting a leftward bias in the direction of first eye movement when people are looking at pictures^1,2^, we analyzed the probability of first fixating the left picture, which corresponded to the total number of first fixations on the picture in the left hemifield divided by the total number of first fixations on the pictures. One-sample t-tests showed that the probability of first fixating on the picture in the left hemifield (M = 51, SE = 3) was significantly greater than .5, t(37) = 3.60, p < .001, d = 0.58.

**Table S1.** Mean (standard deviation) emotional and physical parameters for negative and neutral pictures selected for men and women

|  | Men | | | Women | | |
| --- | --- | --- | --- | --- | --- | --- |
|  | Negative | Neutral | Comparison | Negative | Neutral | Comparison |
| Valence ratings | 2.54 (0.46) | 4.92 (0.28) | t(38.52) = -21.76, p < .001 | 2.49 (0.38) | 4.97 (0.19) | t(33.39) = -28.64, p < .001 |
| Arousal ratings | 5.63 (0.85) | 2.94 (0.59) | t(41.15) = 12.73, p < .001 | 5.68 (0.83) | 2.99 (0.56) | t(40.33) = 13.08, p < .001 |
| Luminance (average) | 93 (31) | 87 (28) | t(45.66) = 0.75, p = .456 | 88 (38) | 84 (35) | t(45.64) = 0.45, p = .655 |
| Luminance (contrast) | 69 (15) | 67 (13) | t(45.36) = 0.492, p = .626 | 67 (17) | 70 (16) | t(45.79) = -0.66, p = .513 |
| Red saturation (average) | 107 (31) | 99 (33) | t(45.86) = 0.91, p = .367 | 99 (41) | 95 (37) | t(45.64) = 0.28, p = .777 |
| Red saturation (contrast) | 76 (15) | 74 (12) | t(43.66) = 0.67, p = .509 | 72 (16) | 77 (16) | t(45.94) = -1.23, p = .227 |
| Green saturation (average) | 75 (29) | 83 (29) | t(45.71) = 0.66, p =.512 | 86 (38) | 81 (36) | t(45.87) = 0.44, p = .660 |
| Green saturation (contrast) | 70 (15) | 68 (15) | t(45.93) = 0.51, p = .616 | 68 (17) | 71 (17) | t(45.90) = -0.58, p = .564 |
| Blue saturation (average) | 75 (29) | 70 (27) | t(45.66) = 0.65, p = .520 | 77 (41) | 67 (35) | t(45.15) = 0.85, p = .401 |
| Blue saturation (contrast) | 65 (18) | 64 (17) | t(45.63) = 0.18, p = .857 | 64 (20) | 64 (18) | t(45.73) = -0.01, p = .997 |
| Spatial frequencies (energy) | | | | | | |
| *Gray*  *Cycles/picture frequency band* |  |  |  |  |  |  |
| 512 to 256 | 123 (114) | 178 (125) | W = 200, p = .070 | 188 (253) | 114 (79) | W = 308, p = .680 |
| 256 to 128 | 1507 (1283) | 2181 (1519) | W = 205, p = .087 | 2195 (2535) | 1427 (927) | W = 316, p = .564 |
| 128 to 64 | 9956 (6421) | 11827 (7764) | W = 258, p = .536 | 11987 (11932) | 8901 (5853) | W = 313, p = .606 |
| 64 to 32 | 63521 (26989) | 57356 (30537) | W = 339, p = .293 | 67265 (58942) | 49813 (30818) | W = 332, p = .364 |
| 32 to 16 | 368232 (135400) | 316093 (164304) | W = 365, p = .112 | 363176 (274437) | 300190 (151115) | W = 316, p = .564 |
| 16 to 8 | 2189010 (802653) | 2052702 (1114087) | W = 337, p = .312 | 2042174 (970741) | 2048469 (1232667) | W = 298, p = .837 |
| 8 to 4 | 13417827 (10820628) | 9900457 (4469756) | W = 355, p = .167 | 11920916 (11413091) | 12212539 (6070704) | W = 243, p = .354 |
| 4 to 2 | 66031649 (47607363) | 66267725 (49153218) | W = 299, p = .821 | 61479587 (52519874) | 71308809 (48323855) | W = 234, p = .266 |
| <2 | 732882898 (385972988) | 643552796 (344249260) | W = 331, p = .375 | 704782930 (453233253) | 669107364 (446325633) | t(45.99) = 0.27, p = .785 |
| *Red*  *Cycles/picture frequency band* |  |  |  |  |  |  |
| 512 to 256 | 123 (114) | 178 (125) | W = 211, p = .112 | 186 (240) | 116 (81) | W = 307, p = .695 |
| 256 to 128 | 1507 (1283) | 2181 (1519) | W = 212, p = .117 | 2178 (2361) | 1447 (940) | W = 320, p = .509 |
| 128 to 64 | 9956 (6421) | 11827 (7764) | W = 260, p = .564 | 12103 (10851) | 9216 (5843) | W = 318, p = .536 |
| 64 to 32 | 63521 (26989) | 57356 (30537) | W = 330, p = .387 | 69928 (53766) | 54589 (30906) | W = 329, p = .398 |
| 32 to 16 | 368232 (135400) | 316093 (164304) | W = 367, p = .103 | 387110 (259209) | 342093 (152673) | W = 299, p = .821 |
| 16 to 8 | 2189010 (802653) | 2052702 (1114087) | W = 327, p = .421 | 2210527 (1010287) | 2396278 (1411857) | W = 278, p = .837 |
| 8 to 4 | 13417827 (10820628) | 9900457 (4469756) | W = 374, p = .076 | 13786398 (12447101) | 14399072 (6220677) | W = 219, p = .155 |
| 4 to 2 | 66031649 (47607363) | 66267725 (49153218) | W = 313, p = .606 | 70721069 (57398457) | 81419148 (54354416) | W = 238, p = .303 |
| <2 | 732882898 (385972988) | 643552796 (344249260) | W = 319, p = .523 | 862933310 (509524575) | 857263880 (515241272) | t(45.54) = 0.74, p = .464 |
| *Green*  *Cycles/picture frequency band* |  |  |  |  |  |  |
| 512 to 256 | 125 (115) | 181 (126) | W = 202, p = .076 | 191 (259) | 117 (81) | W = 307, p = .695 |
| 256 to 128 | 1530 (1288) | 2222 (1546) | W = 208, p = .099 | 2235 (2611) | 1457 (954) | W = 314, p = .592 |
| 128 to 64 | 10196 (6444) | 12200 (8136) | W = 257, p = .523 | 12318 (12478) | 9194 (6208) | W = 312, p = .621 |
| 64 to 32 | 65607 (27926) | 59389 (33891) | W = 345, p = .240 | 69377 (61859) | 51495 (33633) | W = 333, p = .354 |
| 32 to 16 | 380605 (142377) | 324960 (179117) | W = 365, p = .112 | 374413 (286617) | 308259 (167275) | W = 329, p = .398 |
| 16 to 8 | 2265918 (837761) | 2103904 (1205774) | W = 347, p = .224 | 2098452 (996590) | 2106708 (1331277) | W = 302, p = .773 |
| 8 to 4 | 13800247 (11144907) | 10170290 (4983756) | W = 360, p = .138 | 12084128 (11734907) | 12409106 (6485754) | W = 248, p = .410 |
| 4 to 2 | 66687816 (50365129) | 68744320 (53085013) | W = 294, p = .902 | 62505095 (55360821) | 73919740 (51469223) | W = 241, p = .333 |
| <2 | 703830614 (391946387) | 621885755 (356129117) | W = 327, p = .421 | 684129573 (451796300) | 650999185 (459210747) | W = 302, p = .773 |
| *Blue* |  |  |  |  |  |  |
| 512 to 256 | 124 (112) | 171 (117) | W = 210, p = .108 | 186 (254) | 108 (74) | W = 312, p =.621 |
| 256 to 128 | 1509 (1258) | 2063 (1399) | W = 220, p = .161 | 2157 (2549) | 1336 (853) | W = 321, p = .496 |
| 128 to 64 | 10132 (6495) | 10965 (7100) | W = 279, p = .853 | 11819 (12524) | 8149 (5487) | W = 325, p = .446 |
| 64 to 32 | 63409 (29988) | 53220 (29899) | t(46) = 1.18, p = .245 | 64785 (64930) | 44782 (29832) | W = 330, p = .386 |
| 32 to 16 | 363476 (164119) | 292260 (171765) | t(45.91) = 1.47, p = .149 | 344292 (285537) | 263409 (155016) | W = 332, p = .364 |
| 16 to 8 | 2140823 (996721) | 1782990 (873703) | t(45.22) = 1.32, p = .193 | 1902082 (995611) | 1691676 (987826) | t(46.00) = 0.73, p .466 |
| 8 to 4 | 12383414 (8412499) | 9102362 (5038987) | W = 360, p = .138 | 10383493 (8216707) | 10187187 (5812833) | W = 260, p = .564 |
| 4 to 2 | 56868298 (40070328) | 61361718 (45318230) | W = 290, p = .967 | 53042928 (45892590) | 60100444 (40441488) | W = 245, p = .375 |
| <2 | 522128249 (302801867) | 469781886 (320378874) | W = 318, p = .536 | 590504035 (492309254) | 490398706 (444920157) | W = 322, p = .483 |
| Complexity (kilobytes) | 222 (77) | 237 (70) | t(45.59) = -0.71, p = .483 | 218 (84) | 197 (48) | t(40.77) = 0.98, p = .333 |
| Face area (%) | 7.42 (16.08) | 7.77 (17.36) | W = 342, p = 0.252 | 2.77 (7.32) | 3.81 (7.38) | W = 251, p = 0.406 |

*Note. For spatial frequency data,* *energy values correspond to the absolute value of the wavelet coefficients.*

# References

1. Foulsham, T., Gray, A., Nasiopoulos, E. & Kingstone, A. Leftward biases in picture scanning and line bisection: A gaze-contingent window study. *Vision Res.* **78**, 14–25 (2013).

2. Nuthmann, A. & Matthias, E. Time course of pseudoneglect in scene viewing. *Cortex* **52**, 113–119 (2014).
